# Allocating health care resources: a questionnaire experiment on the predictive success of rules

**DOI:** 10.1186/s12939-017-0611-1

**Published:** 2017-06-26

**Authors:** Marlies Ahlert, Lars Schwettmann

**Affiliations:** 1 Department of Economics, Martin Luther University Halle-Wittenberg, D-06099 Halle (Saale), Germany; 2 Institute for Health Economics and Health Care Management, Helmholtz Zentrum München GmbH, Postfach 1129, D-85758 Neuherberg, Germany

**Keywords:** Priority setting for spending, Distribution principles, Classroom experiment, Measure of predictive success, Monotonicity of allocations, Consistency of decision making, Selten’s measure of predictive success

## Abstract

**Background:**

The topic of this paper is related to equity in health within a country. In public health care sectors of many countries decisions on priority setting with respect to treatment of different types of diseases or patient groups are implicitly or explicitly made. Priorities are realized by allocation decisions for medical resources where moral judgments play an important role with respect to goals and measures that should be applied. The aim of this study is to explore the moral intuitions held in the German society related to priorities in medical treatment.

**Methods:**

We use an experimental questionnaire method established in the Empirical Social Choice literature. Participants are asked to make decisions in a sequence of distributive problems where a limited amount of treatment time has to be allocated to hypothetically described patients. The decision problems serve as an intuition pump. Situations are systematically varied with respect to patients’ initial health levels, their ability to benefit from treatment time, and the amount of treatment time available. Subjects are also asked to describe their deliberations. We focus on the acceptance of different allocation principles including equity concepts and utilitarian properties. We investigate rule characteristics like order preservation or monotonicity with respect to resources, severity, or effectiveness. We check the consistency of individual choices with stated reasoning.

**Results:**

The goals and allocation principles revealed show that the moral intuitions held by our experimental subjects are much more complex than the principles commonly applied in health economic theory. Especially, cost-utility principles are rarely applied, whereas the goal of equality of health gain is observed more often. The principle not to leave any patient untreated is very dominant. We also observe the degrees to which extent certain monotonicity principles, known from welfare economics, are followed. Subjects were able to describe their moral judgments in written statements. We also find evidence that they followed their respective intuitions very consistently in their decisions.

**Conclusions:**

Findings of the kind presented in this paper may serve as an important input for the public and political discussion when decisions on priorities in the public health care sector are formed.

**Electronic supplementary material:**

The online version of this article (doi:10.1186/s12939-017-0611-1) contains supplementary material, which is available to authorized users.

## Background

When it comes to the allocation of scarce healthcare resources, decision makers are found to consider a plethora of factors and to apply several often opposing decision criteria of which equity, fairness and effectiveness are the most prominent ones [1]. Both empirically and normatively oriented researchers from various fields identify and characterize outcome-based allocation rules which should or do in fact underlay allocative decisions. In the health economic literature, a growing, concurrent concern for efficiency and equity in deciding on the allocation of healthcare resources has arisen [2], but many more distributive norms are vividly discussed.

In our exploratory study, we shed more light on the acceptance of different allocation principles and typically assumed characteristics of allocation rules applied by laypersons. We distinguish between principles that are related to goals that should be reached by distributing [3, 4], like e.g. equity principles or the utilitarian principle, and properties of sequences of allocations chosen in sets of problems, like e.g. different monotonicity properties. More precisely, we used an established questionnaire design [5, 6] to present a sequence of abstract hypothetical allocation problems of scarce medical resources to participants. Student respondents in the role of a physician had to solve a fixed set of 16 allocation problems. In each situation participants had to distribute a given budget of treatment time among two hypothetical patients who differed with respect to initial health level and ability to benefit from treatment per unit time input, which can be interpreted as time-effectiveness. Subjects were also asked to note their deliberations.

The design of the study enabled us to focus our research on three levels. On the first level, we looked at situations separately and evaluated the predictive power of different allocation principles traditionally analysed and investigated by health economists who usually focus on the trade-off between equity and efficiency [2, 7–9]. One distinctive feature of relevant principles is the “good” to be distributed, i.e. the *distribuendum* [10]. Efficiency concerns usually correspond to the maximization of health gains, but most empirical studies find only weak support for such maximisation behaviour [6, 11–14]. In contrast, equity, and in particular equality principles, may concern several spheres including health gains, outcomes, and medical resources. While support for different egalitarian notions in survey experiments depends on the context [15], equality of health gains is often found to dominate other notions [6, 14, 16]. Additionally, proportionality concepts constitute another alternative [13, 17]. In our study proportionality is related to different abilities to benefit from treatment per unit time input and may focus either on the allocation of resources or gains. Finally, it is regularly observed that participants in experiments trade off allocation principles, identify compromises and often apply conditional rules [18–20]. We have also investigated this phenomenon, which becomes particularly apparent in a content analysis of the subjects’ deliberations.

The application of allocation principles may be accompanied by various additional considerations [21]. First of all, survey respondents often reject the complete exclusion of patients from treatment in micro-level, but not in macro-level contexts [6, 13, 22]. We checked the relevance of the underlying *non-zero principle* for our particular setting. Second, ranking individuals according to the size of the distribuendum is generally found to be an often-applied consideration when allocating scarce resources [23, 24]. We investigated the fulfilment of *order preservation* with respect to health levels in the way that the better-off patient should remain being better off after the allocation of treatment time.

Concerning the second level of our analysis, the set of situations contained pairs specifically constructed to test the fulfilment of three monotonicity axioms under ceteris paribus (c.p.) conditions. First, it has been suggested that variations of the amount of resources available for distribution should influence all individuals in the same direction [25, 26]. Therefore, we considered two hypotheses assuming unchanged initial health levels and time-effectiveness:

- *Strong resource monotonicity:* If the available amount increases (decreases), both patients c.p. receive more (less) treatment time.
- *Weak resource monotonicity*: If the available amount increases (decreases), both patients c.p. receive at least (at most) the same amount of treatment time as before.

Second, severity of illness may be used as an additional criterion for setting priorities in health care [13, 27]. In our experiments, severity was changed by varying initial health levels keeping constant time-effectiveness and amounts of time available:

- *Strong severity monotonicity*: If the initial health level inclines (declines), a patient c.p. receives less (more) treatment time.
- *Weak severity monotonicity*: If the initial health level inclines (declines), a patient c.p. receives at most (at least) as much treatment time as before.
- *Contextual irrelevance of severity*: The allocation of treatment time does not change if a health level changes, c.p.

In general, we use the expression “contextual irrelevance” to indicate that decisions do not change under variations of one specific dimension of the decision problem, c.p.

Third, respondents may react to a change in ability to benefit from treatment per unit time in situations where initial health levels and amounts of time available are constant. How respondents solve the trade-off between efficiency and equity may depend on the contextual parameters. Participants who in a certain context aspire to an efficient allocation should allocate less to a patient if time-effectiveness of her treatment decreases. In contrast, individuals who follow the goal of equality of gains should compensate patients for their decreasing effectiveness. Thus, opposing hypotheses emerged assuming constant initial health levels and amounts of time:

- *Higher effectiveness monotonicity*: If time-effectiveness of their treatment increases (decreases), patients c.p. receive more (less) treatment time.
- *Lower effectiveness monotonicity*: If time-effectiveness of their treatment increases (decreases), patients c.p. receive less (more) treatment time.
- *Contextual irrelevance of effectiveness*: The allocation of treatment time does not change if the effectiveness factor changes, c.p.

At the third level of our analysis, the focus was on individual decision-making regarding the entire sequence of decisions each respondent had to make. Since the order of situations was the same for all respondents, we could compare the development of decision behaviour. Furthermore, experimental studies tend to lack reliable insights into the “real” intentions and motivations of participants. In a somewhat arbitrary “revealed motive” ascription of cognitive processes, distributive choices are often interpreted ad hoc “as if” respondents applied certain distributive principles. This gap can at least partly be closed by incorporating qualitative elements and self-reports. Regardless of some well-known short-comings, such as the fallacy of interpreting the absence of reported motives as absence of such motives [28, 29], corresponding techniques have proven to be an important tool when investigating distributive preferences regarding health care resources [30–33]. Hence, we also asked respondents to verbally describe how they proceeded when making their choices and applied a content analysis. A comparison of the sequence of individual choices and verbal statements facilitated connecting quantitative and qualitative findings and evaluating consistency of answers.

The following sections describe the methodological steps applied, present results of all steps, and discuss them, respectively.

## Methods

### Experiment

After a pre-test with 17 professional health economists, our study was conducted in winter 2012 with 166 German university students attending either their first lecture on health economics in a Master course or a general Bachelor lecture at the law department. The entire questionnaire study was conducted during lecture time. Before answering the questions, respondents were informed that there was no time limit and that participation was entirely voluntary and anonymous. During the experiment one of the authors, three student assistants and, in the law lecture also the lecturer were present. We created an “exam atmosphere” in the way that students were not allowed to talk to each other or to look at the sheets of their neighbours. It took respondents up to 25 min to complete the questionnaire. In each lecture, only two individuals rejected to participate, while in total 162 students agreed.

### The questionnaire

The questionnaire is structured such as to facilitate investigating the validity of the different behavioural hypotheses. In total 16 different allocation problems were presented to each respondent. All hypothetical situations contained information on the amount of treatment time available (*q*) and individual characteristics of two different patients (*i = 1, 2*) who might benefit from the units of time received (*t*_*i*_). In the introduction of the questionnaire (see Additional file 1) participants were informed that patients differed, first, with respect to their current health state (*S*_*i*_), which was measured on a scale reaching from zero (i.e. “death”) to one hundred (i.e. “perfect health”), and, second, with regard to an effectiveness factor (*e*_*i*_), which described their (constant) ability to benefit from each unit of treatment time. Based on these factors a linear “health production function” was assumed:

$$ {H}_i={S}_i+{t}_i\cdotp {e}_i. $$

The simple functional form should make it easier for respondents to understand the implications of different allocations and, in case they could not agree to any of the allocations offered, to make individual proposals. Since the present study intends to consider only a specific set of patients’ attributes it is explicitly stated that nothing is known about the causes of ill health and that patients are of the same age and have the same life expectancy. Furthermore, nothing is said about previous health levels, but it is pointed out that patients remain in the health status reached after treatment for the rest of their lives.

In each situation in the questionnaire (Additional file 1), the problem-specific characteristics are stated at the top of the corresponding table. Below, different allocations of treatment time, resulting health gains and achievable health states are presented. This information is given line-by-line to make it easier for participants to focus on their preferred distribuendum. Table 1 provides an overview of characteristics for all 16 situations, allocations offered and possible principles. Several allocations offered in each situation are based on theoretical considerations – of course without assuming that respondents would be aware of these foundations. Furthermore, all situations contain proposals, which are not theoretically grounded. Additionally, due to the explorative character of the study participants also had the option to make individual proposals and, thereby, apply “non-standard” allocation rules.

**Table 1:** Decision situations, possible principles, and frequencies (N = 162)^a^

Situations					Allocation of time		Possible principle	Frequencies [%]
No.	S_1_	S_2_	e_1_:e_2_	q	Patient 1	Patient 2		
1	40	10	2:1	30	30	0	U	5.6
					25	5	-	1.2
					20	10	PR	11.1
					15	15	ER, PG	20.4
					10	20	EG	44.4
					5	25	-	16.0
					0	30	EH	1.2
2	40	10	2:1	60	30	30	U, ER, PG, PR	10.5
					25	35	-	8.6
					20	40	EG	39.5
					15	45	-	23.5
					10	50	EH	16.7
					5	55	-	1.2
					0	60	-	0.0
3	10	40	2:1	30	30	0	U	2.5
					25	5	-	7.4
					20	10	EH, PR	42.0
					15	15	ER, PG	22.2
					10	20	EG	24.7
					5	25	-	0.6
					0	30	-	0.6
4	10	40	2:1	60	45	15	U	3.1
					40	20	PR	12.3
					30	30	ER, PG, EH	58.0
					20	40	EG	24.7
					10	50	-	0.6
					0	60	-	0.0
					Individual proposal		(35,25)	1.2
5	25	10	2:1	30	30	0	U	3.1
					25	5	-	5.0
					20	10	PR	9.9
					15	15	ER, PG	21.1
					10	20	EG	52.2
					5	25	EH	8.7
					0	30	-	0.0
6	40	25	2:1	30	30	0	U	1.2
					25	5	-	1.9
					20	10	PR	6.8
					15	15	ER, PG	35.8
					10	20	EG	41.4
					5	25	EH	13.0
					0	30	-	0.0
7	70	10	2:1	30	15	15	U, PR, ER, PG	8.0
					10	20	EG	27.2
					5	25	-	43.2
					0	30	EH	21.6
8	30	15	2:1	30	30	0	U	1.2
					25	5	-	2.5
					20	10	PR	8.0
					15	15	ER, PG	37.7
					10	20	EG	39.5
					5	25	EH	11.1
					0	30	-	0.0
9	30	15	2:1	60	35	25	U, PR	6.2
					30	30	ER, PG	11.2
					25	35	-	24.2
					20	40	EG	38.5
					15	45	EH	18.6
					10	50	-	1.2
					0	60	-	0.0
10	40	20	3:1	20	20	0	U	4.9
					15	5	PR	13.0
					10	10	ER, PG	35.8
					5	15	EG	43.2
					0	20	EH	1.9
					Individual proposals		(7,13), (12,8)	1.2
11	40	20	3:1	40	20	20	U, PR, ER, PG	15.5
					15	25	-	23.6
					10	30	EG	46.6
					5	35	EH	13.7
					0	40	-	0.0
					Individual proposal		(24,16)	0.6
12	20	40	3:1	20	20	0	U	4.9
					15	5	PR	20.4
					10	10	ER, PG, EH	54.9
					5	15	EG	19.1
					0	20	-	0.6
13	25	5	3:1	20	20	0	U	13.0
					15	5	PR	14.9
					10	10	ER, PG	20.5
					5	15	EG	48.4
					0	20	EH	3.1
14	25	5	3:1	40	25	15	U, PR	10.5
					20	20	ER, PG	21.6
					15	25	-	14.8
					10	30	EG	40.1
					5	35	EH	13.0
					0	40	-	0.0
15	55	15	3:1	20	15	5	U, PR	5.6
					10	10	ER, PG	23.5
					5	15	EG	62.3
					0	20	EH	8.6
16	55	15	3:1	40	15	25	U, PR, ER, PG	19.1
					10	30	EG	35.2
					5	35	-	42.6
					0	40	EH	2.5
					Individual proposal		(20,20)	0.6

^a^S_1_, S_2_, status quo health levels of patients 1 and 2; e_1_:e_2_, ratio of effectiveness factors of patients 1 and 2; q, available units of treatment time; EG, equality of gains; EH, equality of health levels; ER, equality of resources; PG, proportionality of gains; PR, proportionality of resources; U, utilitarianism. In situations 5, 9, 11, and 13 one answer is missing. In situation 14, the questionnaires differed between both samples, viz. health economics and law lecture: The former did not contain the proposal (15, 25). In the health economics lecture four respondents stated this allocation as a personal proposal

By systematically varying available units of treatment time, current health states or effectiveness factors, we can determine distinct monotonicity conceptions. Compared to the baseline case in situation 1, situations 10 to 16 assume a higher ratio of effectiveness factors. Here, situation 10 serves as a further baseline. In addition, available units of time are increased in six consecutive pairs of situations. Severity differences are varied by changing either one initial health level (situations 5 to 7) or both (situations 8, 9, 15, and 16). In other cases, the difference is left unchanged but levels are either varied by the same extent (situations 13 and 14) or switched (situations 3, 4, and 12).

These systematic variations between situations were subject to two feasibility constraints: First, resulting health levels could not exceed a value of 100 points. Second, to ease the computation for respondents all points should be a multiple of 5 or even 10 so that choices do not depend on the degree of calculative simplicity of solutions. From the content point of view, discussions during the pre-test highlighted the importance of illuminating the entire domain of possible health levels including the boundary areas. Furthermore, it was suggested to offer “intermediate” allocations between specific principles to allow for compromises. The construction of situations accommodated these constraints and suggestions.

Afterwards, participants were asked to give written accounts of their deliberations. Our aim was to stimulate respondents to think about the decisions and to encourage them to express their thoughts in a generalized self-characterization of their decision. The fact that all respondents went through the same sequence of situations enabled us to compare the development of their choices and statements. Finally, participants were asked to provide socio-demographic information including sex, age, field of study, perceived family income ten years ago, expected future income, political orientation, and whether the respondent has already completed a professional training.

### Analysis

Answers are analysed in four major steps. We start by looking at aggregate result. First, the investigation of the proportion of individuals answering in accordance with different allocation principles offers preliminary insights into the general acceptance of competing notions and effects of systematic variations. We apply Selten’s measure of *predictive success* which he developed to evaluate area predictions [34]. The order preservation hypothesis can be interpreted as such an area prediction, since in each situation there are several allocations in accordance with it. For each situation, we calculate the area *a* as the share of allocations fulfilling the property relative to all options offered. The hit rate *r* is defined by the frequency of individual answers in accordance with the property relative to the number of all answers given. The measure of predictive success, *m = r – a,* is an indicator for the quality of the prediction in each situation. One-tailed Binomial tests are applied to evaluate whether *m* is significantly positive.

Second, we turn to individual decisions and pairwise comparisons of selected situations. The fulfilment of the resource, severity, and effectiveness monotonicity hypotheses is investigated by six, four, and two comparisons, respectively. Since the monotonicity hypotheses do not predict unique combinations of choices, but areas, we evaluate the quality of our hypotheses again by using Selten’s measure of predictive success [34]. Here the measure is applied to pairs of choices in specific pairs of situations that can be compared with respect to the monotonicity property under consideration. If for a given context, there are competing area theories, as there are two or three in our analysis of each monotonicity concept, according to Selten’s analysis the one with the higher *m* is the better theory. Again, one-tailed Binomial tests are utilized to assess whether *m* is significantly different from zero. In (Additional file 2: Tables S3 to S7) hit rates, areas of prediction and measures of predictive success for each pair of situations are presented and calculations are explained in detail. In (Additional file 3) areas of prediction used in Tables S4 to S6 of Additional file 2 are constructed in detail.

As a third step, we analyse the data on an individual level and conduct a content analysis of verbal answers of all respondents. First, we developed categories driven by classical theoretical allocation rules. Afterwards, both authors separately organised verbal statements into categories, compared their classifications and discussed potential disagreements. Post-hoc, content not assigned to any category was used to identify new categories with supplementary characteristics of rules. A second round of classifications followed. Finally, a student assistant not yet involved in the process organised all comments into theory-driven and post-hoc categories. Her resulting classifications were compared with those of the authors. Differences were discussed until final agreement was reached.

In a fourth step, we run compatibility tests. For each principle classified in the content analysis, we identify all allocations in each situation, which are in accordance with it. Some of them imply one single allocation in each scenario, for others the corresponding areas of prediction are larger. We then count how often choices in accordance with each principle can actually be observed and calculate actual average hit rates for the total sample. In Table 2, these values are then compared with average hit rates of the subsamples of those respondents who have verbally described the corresponding principle.

**Table 2:** Verbally reported principles and average hit rates^a^

Principle	Verbal reporters: Fraction of respondents describing the principle^a^ (*N* = 155)	Areas of prediction^b^	Actual average hit rates:^b^ Total sample (*N* = 162)	Actual average hit rates:^b^ Only corresponding verbal reporters	Distribution of hits of corresponding verbal reporters (# subjects x hits)
Equality of health levels (EH)	0.1226	0.1802	0.1801	0.3819	3x2, 3x3, 4x5, 1x6, 2x7, 2x8, 1x9, 2x10, 1x16
Equality of health gains (EG)	0.2000	0.1802	0.3913	0.6575	1x3, 3x4, 4x7, 1x8, 3x9, 5x10, 3x11, 1x12, 3x14, 1x15, 6x16
Equality of treatment time (ER)	0.1290	0.1802	0.2594	0.4875	1x2, 2x3, 1x5, 5x6, 3x7, 2x9, 2x10, 2x11, 2x16
Sum-maximization/Utilitarianism (U)	0.0516	0.1802	0.0719	0.1094	5x0, 1x3, 1x4, 1x7
No Exclusion	0.6194	0.7376	0.9481	0.9631	1x12, 3x13, 13x14, 18x15, 61x16
Preference for sicker patient (lower health level)	0.2839	0.5310	0.6256	0.7200	1x4, 2x6, 2x8, 2x9, 6x10, 7x11, 4x12, 11x13, 7x14, 1x15, 1x16
Conditional rules^c^	0.3290	-	-	-	-
Thresholds^c^	0.2129	-	-	-	-

^a^Multiple answers were permitted

^b^The term “average hit rate” denotes the average fraction of actual choices in all situations fitting to the corresponding principle. See Additional file 2: Table S7 for details on the calculation of areas of prediction and actual average hit rates

^c^Conditional rules and rules utilising threshold values are very diverse and do not always and in all situations result in clear allocation proposals

## Results

About 56% of the participants in our experiment were studying at the law department, while 44% were enrolled in Economics, Business Administration, or Business Informatics. Comparing answering patterns of different socio-demographic groups in each situation, we cannot detect any comprehensive influences from individuals’ sex, age, past or future income, professional training, or field of study. With respect to political orientation, we find that in six out of sixteen situations left-wing respondents more often supported solutions in accordance with equality of gains, while right-wing participants more frequently selected solutions leading to equality of resources.

### Accordance with classical allocation principles and order preservation

For each situation, Table 1 reports frequencies of answers for all allocations offered. We focus on major results. Very few individual proposals occurred. First, we look at the frequencies of choices in accordance with classical theoretical conceptions. In 11 out of 16 situations, allocations in accordance with equality of health gains (*EG*) received the highest support. Especially, it is more preferred than the utilitarian principle (*U*). Nevertheless, for example the comparison between situations 1 and 8 reveals that equality of resources (*ER*) is more attractive if initial health levels are more equal. In contrast, equality of health states (*EH*) seems to be especially unattractive in situations where it corresponds to allocations which leave the better-off patient completely untreated.

Probably this concern also made many respondents choose a compromise like (5,25) in situation 7 or (5,35) in situation 16 rather than allocations in accordance with *EG* or *EH*.

In scenarios 3, 4 and 12, where *EG* seems to be less attractive, the worse-off patient 1 is also characterised by a higher effectiveness factor. Consequently, some respondents opted for proposals giving more to patient 1 than *EG* would do. One possible explanation is that participants balanced different arguments in favour of each patient rather than adopting one single principle. This may also explain stronger support for allocations such as (15,45) in situation 2 or (15,25) in situation 11, which do not result from classical theoretical concepts.

Finally, allocations in accordance with proportionality to effectivity factors rarely gained support if they focussed on resources (*PR*), but seemed to be more attractive with respect to health gains (*PG*) which coincides with *ER* due to the linear structure of the health production function.

Additional file 2: Table S3 summarises results on the measures of predictive success of *order preservation*. In 11 out of 14 situations, where order reversals of initial health levels were possible, a great majority kept the original hierarchy and the measure is significantly positive. In contrast, in situations 3, 4 and 12, the measure is significantly negative. We have already elaborated that respondents may be more in favour of *EH* here, which is not in accordance with order preservation in the strict sense.

### Monotonicity properties

Turning to pairwise comparisons of situations, Additional file 2: Table S4 presents results on the measures of predictive success of weak and strong *resource monotonicity*. All measures of predictive success are positive and significant. Hence, there is strong evidence that participants pursued the goal not to reduce the health level of any patient if more treatment time is available. In three of six comparisons, weak monotonicity turns out to be the better area theory, under the other parameter constellations the theory that every patient should gain from higher amounts of treatment time has the highest predictive success. Weak monotonicity seems to yield better predictions in situations where patients are rather asymmetric in terms of current health state and of effectiveness of treatment (see situations 15 and 16).

For the three competing hypotheses, weak and strong *severity monotonicity* and contextual irrelevance of severity, Additional file 2: Table S5 reports the measures of predictive success for four pairwise comparisons of situations. In the first two cases, contextual irrelevance has the highest measure of predictive success, while the measure for strong severity monotonicity is even negative and significant. In contrast, in the latter two pairwise comparisons weak severity monotonicity has the highest predictive success, whereas the measure is even lowest for contextual irrelevance in the very last case. A closer look at the different contexts described by the situations reveals that in the first two pairs of situations the differences between the health states of both patients are reasonably small, whereas the latter two pairs of situations both contain situation 1 where the health state of patient 1 compared to patient 2 is remarkably higher. Hence, we are prepared to say that many respondents do not differentiate between states of severity if these are rather low and (thus) of similar size, whereas larger severity differences lead to some concern for severity. This finding will also be confirmed in our content analysis.

With respect to changes of effectiveness factors, the results summarised in Additional file 2: Table S6 reveal that all three hypotheses are of low quality in the two pairs of situations compared. Higher *effectiveness monotonicity* turns out to have negative measures of predictive success and therefore has to be rejected in both cases; lower effectiveness monotonicity has in both cases a predictive success close to zero. The best theory here seems to be contextual irrelevance of effectiveness.

### Content analysis

One hundred fifty five out of 162 participants (96%) also provided verbal statements on allocation rules applied. In Table 2 we distinguish between classical theory-driven principles, that is different equality notions or utilitarianism, and further principles identified in the explorative part of the analysis. The second column reports how frequently each principle was mentioned. Note, that very few respondents used proportionality arguments. Furthermore, those people had difficulties to clearly signal what it was that they wanted to allocate proportionally so that corresponding principles are omitted.

As expected from the quantitative results, *EG* has been mentioned more often than other ideas, while the maximisation of sums of health points received least support. Some respondents even mentioned utilitarian concerns but also explained why they departed from the related rule. Here is a typical example for a corresponding statement:

“In general, it was important for me that the cumulative health state is rather high than low. However, I have never chosen the maximum to avoid that one person is particularly worse off compared to the other person.”

In total, these classical concepts appear in less than half of all verbal statements.

Regarding non-classical categories, more than 60% of all comments stated that no one should be completely excluded from treatment and, thus, supported the non-zero principle. Typical qualitative terms include “treat everybody”, “both patients”, and “no exclusion”.

Another 28% of the respondents expressed a preference for the worse-off patient without necessarily demanding equality:

“In general, I prefer equality of time. But if a person is in good health, the other person should receive more.”

This statement also belongs to the group of conditional rules. Every third respondent combined at least two principles and defined conditions for a switch from one principle to another by using terms such as “but”, “if”, or “however”. Finally, several participants specified threshold values either to identify the aforementioned switching point or to develop a separate rule:

“Then the aim was to reach a health level of 50. […] By this, life would be reasonably liveable.”

The results in Table 2 show that in general non-classical principles were mentioned more frequently than the classical concepts of equality or utilitarianism. Although we only asked respondents to describe the rules they developed, some stated their main motives. At least 28 respondents pronounced that they wanted to find a “fair” or “just” solution, while other motives were rarely mentioned. Hence, in our study justice concerns seem to be a prominent motivation.

### Compatibility checks

A common characteristic of the four classical categories *EH*, *EG*, *ER*, and *U* is that in each situation considered each of them determines a single solution. Consequently, areas of prediction are identical as stated in the third column of Table 2 (see Additional file 2: Table S7, for details). In contrast, the concepts “no exclusion” and “preference for the sicker patient” constrain the list of compatible allocations rather than identifying a specific answer. Thus, it can be expected that more choices will fulfil these notions. Finally, several conditional rules and threshold values have been proposed, but they differ remarkably, so that joint statements about the rate of fulfilment in all situations are hardly possible.

The results reported in columns three to five of Table 2 facilitate evaluating the compatibility of verbal statements and choices in all situations. Some of the actual average hit rates for the entire sample are higher than the values of the corresponding areas of prediction. Especially the strong support for the “no exclusion” idea is visible from the actual average hit rates of about 95%. However, the differences also concern *EG* and, to a lesser degree, *ER* and “preference for the sicker patient”. Hence, these considerations had a visible influence on some respondents’ choices. If we focus only on subjects who explicitly described the corresponding principle, their actual average hit rates in column 5 are even higher for all principles compared to the total sample, and also to the corresponding area of prediction. The only exception is sum-maximisation. In summary, respondents have followed their verbally reported rules rather consistently.

## Discussion

From our point of view, empirical work such as the present questionnaire study can be used to elicit the variety of different allocation intuitions and to identify characteristics of feasible and acceptable solutions for distributive problems in health care provision. The results of the study corroborate the pluralism and heterogeneity in basic conceptions of resource allocation in particular in medical resource allocation.

A unique principle applied by all respondents and in all situations does not exist. Instead, we observe a variety of different allocation principles. Nevertheless, aggregate frequencies of choices and especially verbal statements suggest that for many respondents health gain egalitarianism was the most important classical principle in several situations, while equality of health received more support if the worse-off patient also had a higher ability to benefit. These results confirm observations from several previous studies [6, 14, 16]. In contrast, health maximisation has regularly been rejected, which is also in line with previous results cited in the introduction. In our contexts, this is especially due to the fact that many respondents wanted to avoid any complete exclusion of patients from treatment.

Furthermore, we identified two specific *compensation motives*. On the one hand, several respondents withdrew from focussing on higher effectiveness and compensated for lower ability to benefit. On the other hand, many participants explained that they were prepared to give more to a patient if this person was *clearly* worse off. Our results with respect to severity monotonicity endorse this effect. Hence, we conclude that different notions of effectiveness monotonicity are moderated by severity differences.

However, compensation motives also seem to have their limits. Despite stronger support for the worse-off patient, many respondents abstained from allocations in accordance with health egalitarianism in most situations. Consequently, order preservation with respect to health status before and after treatment has been fulfilled by an overwhelming majority of participants in almost all situations. This concept has already been identified as an important characteristic of allocation rules in different contexts [23, 24], but it is remarkable that it is also relevant in health care allocation problems.

As regularly observed in empirical studies [18–20], many people report allocation rules that express compromises between competing allocation principles. The specific construction and systematic variation of situations allowed for a greater variety of different concerns and intermediate positions. In line with some earlier findings [21], respondents preferred stronger support for worse-off recipients of care but did not try to equalise health levels. They applied conditional rules, defined threshold values, or violated order preservation only if several arguments spoke in favour of supporting the worse-off patient. Future theoretical models should take hierarchies of principles and conditional rules into account and will have to deal with more sophisticated requirements for their application as revealed by our participants.

The content analysis forms an important complementary element to the decisions in the single situations. The high proportion of respondents who gave answers, often with long and detailed elaborations, together with the astonishing consistency between their described allocation rules and previous choices, make us confident that participants took their tasks seriously.

The present study is subject to potential limitations. First, due to the simple linear structure of the health production function, distinct principles led to identical allocations in some of our situations. Simplified allocation problems only allow for a certain set of allocation principles, so that other prominent principles might be ignored by design [9, 21]. However, to keep the calculations manageable for respondents this seems a price worth paying. Furthermore, remaining variations between solutions and among situations across the entire domain of possible health levels seem to be sufficient to differentiate among principles, to allow for compromises, and to examine the relevance of allocation principles also for very high or low levels. Second, all respondents answered decision problems in the same order. Obviously, there may be ordering effects in that previous answers influenced later responses. However, with regard to our aim to interpersonally compare consistency of sequences of decisions to verbal statements it was important to let all respondents work through the same series of problems in exactly the same order. Third, the sample consisted only of students who, moreover, came from just two different fields of study. In general, experts may be biased by prejudices or conflicts of interest, while representative samples of the general public may be more well-meaning but less able to state their intuitions coherently [35, 36]. With respect to the allocation of health care resources, members of the general public often tend to think about trade-offs between abstract alternatives in terms of concrete examples, thereby, solely rely on intuitions rather than well-defined abstract principles [37]. Therefore, students are often chosen as a compromise, as they are regularly seen as better able to investigate numerical decision problems analytically and less error-prone than members of the general public, while their intuitions are less biased compared to experts.

Fourth, we have presented micro-justice contexts, in which a decision maker was asked to distribute a resource between two single patients. Since these patients are described in a very abstract and non-personal manner, they could also be regarded as representatives for larger groups. Nevertheless, the general question arises as to whether results of micro-justice investigations are relevant for large-scale problems. Clearly, consistency of decisions between the micro and the macro level is an important requirement for health-care rationing [38, 39]. This is especially the case in a statutory health insurance system, where each patient is eligible to receive the same treatment as other patients with the same diagnosis. In practice, medical guidelines are a response to this demand.

The position of the decision maker might be a further matter of concern [40, 41]. Impartiality and sympathy are preconditions for normative judgements, whereas personal involvement is likely to trigger material or immaterial self-interest. From our point view, the position of the physician in the questionnaire is in between these two pure positions. On the one hand, despite the hypothetical character of the situations respondents may have felt obliged to help both patients due to professional ethics or because they imagine to stand at the bed-side of the patients. On the other hand, patients are described in a very abstract way. The questionnaire only states numerical information being relevant for the application of different allocation principles considered. Hence, at least there is no direct real or hypothetical partiality and, indeed, many respondents mentioned ‘fairness’ and ‘justice’ as their main motives.

## Conclusions

The topic of this paper is related to equity in health within a country. Health policy decision makers in almost all developed countries must cope with the fact that the growing usefulness of healthcare technologies increases the demand for healthcare services such that scarcity becomes tangible. Criteria for priority setting and rationing of healthcare resources with respect to treatment of different types of diseases or patient groups are implicitly or explicitly made. This implies that priorities are realized by allocation decisions where medical resources are distributed. Independently of which institution may make these decisions in a publicly financed healthcare system, being it the group of medical doctors or a political decision, these criteria should be chosen transparently and discussed in society. Thus, public preferences play an important role in such a discourse.

The aim of our study is to explore the moral intuitions held by non-expert participants related to priorities in medical treatment. To observe the goals and moral attitudes when allocating scarce medical resources, we use an experimental questionnaire method established in the Empirical Social Choice literature where hypothetical decision problems presented serve as an intuition pump [36]. The goals and allocation principles revealed show that the moral intuitions held by our experimental subjects are much more complex than the principles commonly applied in health economic theory. Especially, cost-utility principles are rarely applied, whereas the goal of equality of health gain is observed more often. The principle not to leave any patient untreated is very dominant. We also observe the degrees to which extent certain monotonicity principles, known from welfare economics, are followed. We find evidence that subjects followed their respective intuitions very consistently in their decisions and were able to verbally specify the allocation rules applied.

Thus, overall our exploratory experimental findings reveal insights which allocation principles may be accepted in an abstract context. Results of that kind may then serve as an important input for the public and political discussion when decisions on priorities in the public health care sector are formed [42].

## Additional files

Additional file 1: The Questionnaire. (PDF 532 kb)

Additional file 2: **Table S3.** Order preservation. **Table S4.** Weak and strong resource monotonicity. **Table S5.** Weak and strong severity monotonicity. **Table S6.** Effectiveness monotonicity. **Table S7.** Principles and compatibilities. (PDF 332 kb)

Additional file 3: **Table S4.**a. Pairs of allocations fulfilling weak and/or strong resource monotonicity – Situations 1 and 2. **Table S4.**b. Pairs of allocations fulfilling weak and/or strong resource monotonicity – Situations 3 and 4. **Table S4.**c. Pairs of allocations fulfilling weak and/or strong resource monotonicity – Situations 8 and 9. **Table S4.**d. Pairs of allocations fulfilling weak and/or strong resource monotonicity – Situations 10 and 11. **Table S4.**e. Pairs of allocations fulfilling weak and/or strong resource monotonicity – Situations 13 and 14. **Table S4.**f. Pairs of allocations fulfilling weak and/or strong resource monotonicity – Situations 15 and 16. **Table S5.**a. Pairs of allocations fulfilling weak and/or strong severity monotonicity – Situations 1 and 5. **Table S5.**b. Pairs of allocations fulfilling weak and/or strong severity monotonicity – Situations 1 and 6. **Table S5.**c. Pairs of allocations fulfilling weak and/or strong severity monotonicity – Situations 1 and 7. **Table S5.**d. Pairs of allocations fulfilling weak and/or strong severity monotonicity – Situations 5 and 7. **Table S6**.a. Pairs of allocations fulfilling effectiveness monotonicity – Situations 1 and 3. **Table S6.**b. Pairs of allocations fulfilling effectiveness monotonicity – Situations 10 and 12. (PDF 446 kb)

## Abbreviations

c.p.: Ceteris paribus
EG: Equality of health gains
EH: Equality of health states
ER: Equality of resources
PG: Proportionality of health gains
PR: Proportionality of resources
U: Utilitarian principle
