# Supplementary material for: Allocating health care resources: a questionnaire experiment on the predictive success of rules
Source: Int J Equity Health. 2017 Jun 26;16:112. doi: 10.1186/s12939-017-0611-1 (PMC5485565; doi:10.1186/s12939-017-0611-1)
Supplement: Supplementary file 1 — The Questionnaire. (PDF 532 kb) [file 12939_2017_611_MOESM1_ESM.pdf]

Allocating Health Care Resources:
A Questionnaire Experiment on the Predictive Success of Rules

Additional file 1: The Questionnaire

Survey in lecture XXX

Instructions

In the following survey, you will face simplified medical decision problems. In each situation, you are asked to allocate treatment time among hypothetical patients. The situations have been constructed for this survey and have no concrete background. There is no right or wrong answer. Of course, the survey is anonymous. Thank you very much for your participation.

Put yourself in the position of a physician. In each of the following decision problems you have a fixed budget of treatment time at your disposal. You can allocate this treatment time among at most two patients. Male and female patients are not distinguished linguistically. In each situation, a new pair of patients and a new time budget is considered.

Suppose that both patients are of the same age and have a life-expectancy of another 20 years. Both patients have different diseases, which harm their health states. The reasons for the diseases are not known.

Irrespective of their health status the patients respond differently to the treatment. Consequently, each unit of treatment time has a different effectivity for both patients. After the elapse of the available time a further improvement of the health states is not possible; the patients remain in their health state reached for the rest of their life.

In each decision situation, the following information is available:

First, the **current health states** are given in points. The health status is measured on a point scale from 0 (i.e. "death") to 100 (i.e. "perfect health").

Second, for each patient an **effectivity factor** is stated. The factor measures in points the improvement of health in points which a patient gains from each unit of treatment time obtained. In medical terms, it concerns valuable prognostic information on the treatment success. The factor of a patient remains constant for each unit of time received.

Third, the available **budget of treatment time** is stated in each situation.

To help you with your decision in each situation, allocation proposals are offered. For each proposal, the resulting health gains measured in points are stated. Furthermore, starting from the current health states, for both patients' health levels achieved after the allocation of time and measured in points are given.

Please tick in each situation the allocation, which most likely corresponds to your preferences. Only if you cannot agree to any of the proposals, we ask you to state your own proposal.

**Situation 1**	Patient 1	Patient 2
Current health state	40	10
Effectivity factor	2	1
Available units of treatment time	30	

Patient	1	2	1	2	1	2	1	2	1	2	1	2	1	2	Own proposal 1	Own proposal 2
Allocation of time	30	0	25	5	20	10	15	15	10	20	5	25	0	30		
Health gain (in points)	60	0	50	5	40	10	30	15	20	20	10	25	0	30		
Health state after the allocation of time	100	10	90	15	80	20	70	25	60	30	50	35	40	40		
Your favoured allocation	O		O		O		O		O		O		O			

**Situation 2**	Patient 1	Patient 2
Current health state	40	10
Effectivity factor	2	1
Available units of treatment time	60	

Patient	1	2	1	2	1	2	1	2	1	2	1	2	1	2	Own proposal 1	Own proposal 2
Allocation of time	30	30	25	35	20	40	15	45	10	50	5	55	0	60		
Health gain (in points)	60	30	50	35	40	40	30	45	20	50	10	55	0	60		
Health state after the allocation of time	100	40	90	45	80	50	70	55	60	60	50	65	40	70		
Your favoured allocation	O		O		O		O		O		O		O			

**Situation 3**	Patient 1	Patient 2
Current health state	10	40
Effectivity factor	2	1
Available units of treatment time	30	

Patient	1	2	1	2	1	2	1	2	1	2	1	2	1	2	Own proposal 1	Own proposal 2
Allocation of time	30	0	25	5	20	10	15	15	10	20	5	25	0	30		
Health gain (in points)	60	0	50	5	40	10	30	15	20	20	10	25	0	30		
Health state after the allocation of time	70	40	60	45	50	50	40	55	30	60	20	65	10	70		
Your favoured allocation	O		O		O		O		O		O		O			

**Situation 4**	Patient 1	Patient 2
Current health state	10	40
Effectivity factor	2	1
Available units of treatment time	60	

Patient	1	2	1	2	1	2	1	2	1	2	1	2	Own proposal 1	Own proposal 2
Allocation of time	45	15	40	20	30	30	20	40	10	50	0	60		
Health gain (in points)	90	15	80	20	60	30	40	40	20	50	0	60		
Health state after the allocation of time	100	55	90	60	70	70	50	80	30	90	10	100		
Your favoured allocation	O		O		O		O		O		O			

**Situation 5**	Patient 1	Patient 2
Current health state	25	10
Effectivity factor	2	1
Available units of treatment time	30	

Patient	1	2	1	2	1	2	1	2	1	2	1	2	1	2	Own proposal 1	Own proposal 2
Allocation of time	30	0	25	5	20	10	15	15	10	20	5	25	0	30		
Health gain (in points)	60	0	50	5	40	10	30	15	20	20	10	25	0	30		
Health state after the allocation of time	85	10	75	15	65	20	55	25	45	30	35	35	25	40		
Your favoured allocation	O		O		O		O		O		O		O			

**Situation 6**	Patient 1	Patient 2
Current health state	40	25
Effectivity factor	2	1
Available units of treatment time	30	

Patient	1	2	1	2	1	2	1	2	1	2	1	2	1	2	Own proposal 1	Own proposal 2
Allocation of time	30	0	25	5	20	10	15	15	10	20	5	25	0	30		
Health gain (in points)	60	0	50	5	40	10	30	15	20	20	10	25	0	30		
Health state after the allocation of time	100	25	90	30	80	35	70	40	60	45	50	50	40	55		
Your favoured allocation	O		O		O		O		O		O		O			

**Situation 7**	Patient 1	Patient 2
Current health state	70	10
Effectivity factor	2	1
Available units of treatment time	30	

Patient	1	2	1	2	1	2	1	2	Own proposal 1	Own proposal 2
Allocation of time	15	15	10	20	5	25	0	30		
Health gain (in points)	30	15	20	20	10	25	0	30		
Health state after the allocation of time	100	25	90	30	80	35	70	40		
Your favoured allocation	O		O		O		O			

**Situation 8**	Patient 1	Patient 2
Current health state	30	15
Effectivity factor	2	1
Available units of treatment time	30	

Patient	1	2	1	2	1	2	1	2	1	2	1	2	1	2	Own proposal 1	Own proposal 2
Allocation of time	30	0	25	5	20	10	15	15	10	20	5	25	0	30		
Health gain (in points)	60	0	50	5	40	10	30	15	20	20	10	25	0	30		
Health state after the allocation of time	90	15	80	20	70	25	60	30	50	35	40	40	30	45		
Your favoured allocation	O		O		O		O		O		O		O			

**Situation 9**	Patient 1	Patient 2
Current health state	30	15
Effectivity factor	2	1
Available units of treatment time	60	

Patient	1	2	1	2	1	2	1	2	1	2	1	2	1	2	Own proposal 1	Own proposal 2
Allocation of time	35	25	30	30	25	35	20	40	15	45	10	50	0	60		
Health gain (in points)	70	25	60	30	50	35	40	40	30	45	20	50	0	60		
Health state after the allocation of time	100	40	90	45	80	50	70	55	60	60	50	65	30	75		
Your favoured allocation	O		O		O		O		O		O		O			

**Situation 10**	Patient 1	Patient 2
Current health state	40	20
Effectivity factor	3	1
Available units of treatment time	20	

Patient	1	2	1	2	1	2	1	2	1	2	Own proposal 1	Own proposal 2
Allocation of time	20	0	15	5	10	10	5	15	0	20		
Health gain (in points)	60	0	45	5	30	10	15	15	0	20		
Health state after the allocation of time	100	20	85	25	70	30	55	35	40	40		
Your favoured allocation	O		O		O		O		O			

**Situation 11**	Patient 1	Patient 2
Current health state	40	20
Effectivity factor	3	1
Available units of treatment time	40	

Patient	1	2	1	2	1	2	1	2	1	2	Individual proposal 1	Individual proposal 2
Allocation of time	20	20	15	25	10	30	5	35	0	40		
Health gain (in points)	60	20	45	25	30	30	15	35	0	40		
Health state after the allocation of time	100	40	85	45	70	50	55	55	40	60		
Your favoured allocation	O		O		O		O		O			

**Situation 12**	Patient 1	Patient 2
Current health state	20	40
Effectivity factor	3	1
Available units of treatment time	20	

Patient	1	2	1	2	1	2	1	2	1	2	Own proposal 1	Own proposal 2
Allocation of time	20	0	15	5	10	10	5	15	0	20		
Health gain (in points)	60	0	45	5	30	10	15	15	0	20		
Health state after the allocation of time	80	40	65	45	50	50	35	55	20	60		
Your favoured allocation	O		O		O		O		O			

**Situation 13**	Patient 1	Patient 2
Current health state	25	5
Effectivity factor	3	1
Available units of treatment time	20	

Patient	1	2	1	2	1	2	1	2	1	2	Own proposal 1	Own proposal 2
Allocation of time	20	0	15	5	10	10	5	15	0	20		
Health gain (in points)	60	0	45	5	30	10	15	15	0	20		
Health state after the allocation of time	85	5	70	10	55	15	40	20	25	25		
Your favoured allocation	O		O		O		O		O			

**Situation 14**	Patient 1	Patient 2
Current health state	25	5
Effectivity factor	3	1
Available units of treatment time	40	

Patient	1	2	1	2	1	2	1	2	1	2	1	2	Own proposal 1	Own proposal 2
Allocation of time	25	15	20	20	15	25	10	30	5	35	0	40		
Health gain (in points)	75	15	60	20	45	25	30	30	15	35	0	40		
Health state after the allocation of time	100	20	85	25	70	30	55	35	40	40	25	45		
Your favoured allocation	O		O		O		O		O		O			

**Situation 15**	Patient 1	Patient 2
Current health state	55	15
Effectivity factor	3	1
Available units of treatment time	20	

Patient	1	2	1	2	1	2	1	2	Own proposal 1	Own proposal 2
Allocation of time	15	5	10	10	5	15	0	20		
Health gain (in points)	45	5	30	10	15	15	0	20		
Health state after the allocation of time	100	20	85	25	70	30	55	35		
Your favoured allocation	O		O		O		O			

**Situation 16**	Patient 1	Patient 2
Current health state	55	15
Effectivity factor	3	1
Available units of treatment time	40	

Patient	1	2	1	2	1	2	1	2	Own proposal 1	Own proposal 2
Allocation of time	15	25	10	30	5	35	0	40		
Health gain (in points)	45	25	30	30	15	35	0	40		
Health state after the allocation of time	100	40	85	45	70	50	55	55		
Your favoured allocation	O		O		O		O			

Please describe briefly how you proceeded in general when making your decisions in the different situations. On which information did you focus in particular? Did you try to reach a certain goal with your allocations? Did you develop a rule for your decisions, and if yes, how would you describe this rule?

Finally, we ask you for some personal information. Please keep in mind that the questionnaire is anonymous and your answers are only used for scientific purposes.

1. Gender: O female O male
2. Age: ________ years
3. Federal state where you received your qualification for university entrance:
   ____
4. Have you already finished a professional training (including military or civilian service or voluntary year of social service) or done a job?

   O No. O Yes, I have worked as
   ____
5. First current study course: ______________________________ Semesters at university: ___

   Second current study course (if applicable): _______________ Semesters at university: ___
6. How would you rate your family's income ten years ago?

   very poor O —— O —— O —— O —— O —— O —— O very rich
7. How would you rate your income prospects in ten years?

   very low O —— O —— O —— O —— O —— O —— O very high
8. How would you rate your political view?

   far to the left O —— O —— O —— O —— O —— O —— O far to the right

Thank you very much for your participation.
