# Supplementary material for: Allocating health care resources: a questionnaire experiment on the predictive success of rules
Source: Int J Equity Health. 2017 Jun 26;16:112. doi: 10.1186/s12939-017-0611-1 (PMC5485565; doi:10.1186/s12939-017-0611-1)
Supplement: Supplementary file 2 — Table S3. Order preservation. Table S4. Weak and strong resource monotonicity. Table S5. Weak and strong severity monotonicity. Table S6. Effectiveness monotonicity. Table S7. Principles and compatibilities. (PDF 332 kb) [file 12939_2017_611_MOESM2_ESM.pdf]

Allocating Health Care Resources:
A Questionnaire Experiment on the Predictive Success of Rules

Additional file 2: Additional Tables S3 to S7

Remark: For those who are interested in the detailed calculation of hit rates and areas of prediction for each pair of situations in Tables S4 to S6, we provide supplementary material in Additional file 3

Table S3: Order preservation [a]

Situation	N	Hit rate: Fraction of actual fulfilment (r)	Area of prediction (a) [b]	Measure of predictive success ($m = r - a$) [c]
1	162	0.9877	0.8571	0.1305**
2	162	0.8210	0.5714	0.2496**
3	162	0.4815	0.5714	-0.0899*
4	160	0.2531	0.5000	-0.2469**
5	161	0.9130	0.7143	0.1988**
6	162	0.8704	0.7143	0.1561**
8	162	0.8889	0.7143	0.1746**
9	161	0.8012	0.5714	0.2298**
10	160	0.9815	0.8000	0.1815**
11	160	0.8634	0.6000	0.2634**
12	162	0.1975	0.4000	-0.2025**
13	161	0.9689	0.8000	0.1689**
14	162	0.8704	0.6667	0.2037**
16	161	0.9753	0.7500	0.2253**

[a] Individual proposals are omitted. In situations 7 and 15, order preservation is always fulfilled due to the construction of the situations.

[b] Area of prediction (a) is based on the proportion of those allocations offered in the questionnaire which are in accordance to order preservation. Example: In situation 1, 6 out of 7 allocations in the questionnaire leave patient 1 better off than patient 2.

[c] One-tailed Binomial tests for the difference between hit rate (r) and area of prediction (a): levels of significance * p<0.05, ** p<0.01.

Table S4: Weak and strong resource monotonicity

		Weak resource monotonicity			Strong resource monotonicity		
Situations compared	Sample size [a]	Hit rate: Fraction of actual fulfilment (r_w)	Area of prediction (a_w) [b]	Measure of predictive success ($m_w = r_w - a_w$) [c]	Hit rate: Fraction of actual fulfilment (r_s)	Area of prediction (a_s) [b]	Measure of predictive success ($m_s = r_s - a_s$) [c]
Sit.1, Sit.2	162	0.9321	0.5714	0.3607**	0.7469	0.4082	0.3388**
Sit.3, Sit.4	160	0.9875	0.5952	0.3923**	0.9250	0.4286	0.4964**
Sit.8, Sit.9	161	0.9938	0.6531	0.3407**	0.9379	0.4898	0.4481**
Sit.10, Sit.11	159	0.9497	0.6000	0.3497**	0.5975	0.3600	0.2375**
Sit.13, Sit.14	161	0.9006	0.6333	0.2673**	0.7143	0.4000	0.3143**
Sit.15, Sit.16	161	0.9379	0.6250	0.3129**	0.4534	0.3750	0.0784*

[a] Individual proposals are omitted.

[b] Area of prediction (a) is based on the proportion of those allocations offered in the questionnaire which are in accordance to weak or strong resource monotonicity.

[c] One-tailed Binomial tests for the difference between hit rate (r) and area of prediction (a): levels of significance * p<0.05, ** p<0.01.

Table S5: Weak and strong severity monotonicity

Situations compared	Sample size	Weak severity monotonicity: Hit rate: Fraction of actual fulfilment (r_w)	Weak severity monotonicity: Area of prediction (a_w)	Weak severity monotonicity: Measure of predictive success ($r_w - a_w$)	Strong severity monotonicity: Hit rate: Fraction of actual fulfilment (r_s)	Strong severity monotonicity: Area of prediction (a_s)	Strong severity monotonicity: Measure of predictive success ($r_s - a_s$)	Contextual irrelevance of severity: Hit rate: Fraction of actual fulfilment (r_i)	Contextual irrelevance of severity: Area of prediction (a_i)	Contextual irrelevance of severity: Measure of predictive success ($r_i - a_i$)
**Sit. 1** (S_1=40, S_2=10; e_1:e_2= 2:1) **Sit. 5** (S_1=25, S_2=10; e_1:e_2= 2:1)	161	0.8012	0.5714	0.2298**	0.3354	0.4286	-0.0932*	0.4658	0.1429	0.3230**
**Sit. 6** (S_1=40, S_2=25; e_1:e_2= 2:1) **Sit.1** (S_1=40, S_2=10; e_1:e_2= 2:1)	162	0.7593	0.5714	0.1878**	0.2963	0.4286	-0.1323**	0.4630	0.1429	0.3201**
**Sit. 1** (S_1=40, S_2=10; e_1:e_2= 2:1) **Sit. 7** (S_1=70, S_2=10; e_1:e_2= 2:1)	162	0.9568	0.7857	0.1711**	0.6975	0.6429	0.0547	0.2593	0.1429	0.1164**
**Sit. 5** (S_1=25, S_2=10; e_1:e_2= 2:1) **Sit. 7** (S_1=70, S_2=10; e_1:e_2= 2:1)	161	0.9627	0.7857	0.1770**	0.7640	0.6429	0.1211**	0.1988	0.1429	0.0559*

Note: S_1, S_2, status quo health levels of patients 1 and 2; e_1, e_2, effectiveness factors of patients 1 and 2. Individual proposals are omitted. Area of prediction (a) is based on the proportion of those allocations offered in the questionnaire which are in accordance to weak or strong severity monotonicity.

One-tailed Binomial tests for the difference between hit rate and area of prediction: levels of significance * $p<0.05$, ** $p<0.01$.

Table S6: Effectiveness monotonicity

		Focus on higher effectiveness			Focus on lower effectiveness			Contextual irrelevance of effectiveness		
Situations compared [a]	Sample size	Hit rate: Fraction of actual fulfilment (r_h)	Area of prediction (a_h)	Measure of predictive success ($r_h - a_h$)	Hit rate: Fraction of actual fulfilment (r_l)	Area of prediction (a_l)	Measure of predictive success ($r_l - a_l$)	Hit rate: Fraction of actual fulfilment (r_n)	Area of prediction (a_n)	Measure of predictive success ($r_n - a_n$)
**Sit. 1** (S_1=40, S_2=10; e_1:e_2= 2:1) **Sit. 3** (S_1=40, S_2=10; e_1:e_2= 2:1)	162	0.2531	0.4286	-0.1755**	0.4815	0.4286	0.0529	0.2654	0.1429	0.1226**
**Sit. 10** (S_1=40, S_2=20; e_1:e_2= 3:1) **Sit. 3** (S_1=20, S_2=40; e_1:e_2= 3:1)	160	0.2563	0.4000	-0.1438**	0.4688	0.4000	0.0688*	0.2750	0.2000	0.0750*

S_1, S_2, status quo health levels of patients 1 and 2; e_1, e_2, effectivity factors of patients 1 and 2. Individual proposals are omitted. Area of prediction (a) is based on the proportion of those allocations offered in the questionnaire which are in accordance to both versions of effectivity monotonicity. One-tailed Binomial tests for the difference between hit rate (r) and area of prediction (a): levels of significance * $p<0.05$, ** $p<0.01$.

Table S7: Principles and compatibilities

**Situation**	1	2	3	4	5	6	7	8	9	10	11	12	13	14	15	16
# of answers offered in the questionnaire (individual proposal are omitted)	7	7	7	6	7	7	4	7	7	5	5	5	5	6	4	4
**Principles:**																
Equality of health levels (EH)	7	5	3	3	6	6	4	6	5	5	4	3	5	5	4	4
Equality of health gains (EG)[a]	5	3	5	4	5	5	2	5	4	4	3	4	4	4	3	2
Equality of treatment time (ER)[a]	4	1	4	3	4	4	1	4	2	3	1	3	3	2	2	1
Sum-maximization/ Utilitarianism (U)	1	1	1	1	1	1	1	1	1	1	1	1	1	1	1	1
No Exclusion	2-6	1-6	2-6	1-5	2-6	2-6	1-3	2-6	1-6	2-4	1-4	2-4	2-4	1-5	1-3	1-3
Preference for sicker patient (lower health level)	5-7	2-7	1-3	1,2	5-7	5-7	2-4	5-7	3-7	4,5	2-5	1,2	4,5	3-6	3,4	1-4

Answers are numbered consecutively as they appear in the questionnaire from left (highest feasible amount for patient 1) to right (highest amount for patient 2) or top down for each situation in Table 1 in the text. Matrix cells show for each situation the answers which are compatible with the principle.

Example: Calculating the area of prediction for the principle “no exclusion”:

$$[5/7 + 6/7 + 5/7 + 5/6 + 5/7 + 5/7 + 3/4 + 5/7 + 6/7 + 3/5 + 4/5 + 3/5 + 3/5 + 5/6 + 3/4 + 3/4] / 16 = 0.7376$$

[a] In situations 7 and 15, equality of health levels is not feasible. In situation 16, equality of treatment time is not reasonable. In these situations, the equality principles are replaced by corresponding leximin principles.
