# Supplementary material for: Allocating health care resources: a questionnaire experiment on the predictive success of rules
Source: Int J Equity Health. 2017 Jun 26;16:112. doi: 10.1186/s12939-017-0611-1 (PMC5485565; doi:10.1186/s12939-017-0611-1)
Supplement: Supplementary file 3 — Table S4.a. Pairs of allocations fulfilling weak and/or strong resource monotonicity – Situations 1 and 2. Table S4.b. Pairs of allocations fulfilling weak and/or strong resource monotonicity – Situations 3 and 4. Table S4.c. Pairs of allocations fulfilling weak and/or strong resource monotonicity – Situations 8 and 9. Table S4.d. Pairs of allocations fulfilling weak and/or strong resource monotonicity – Situations 10 and 11. Table S4.e. Pairs of allocations fulfilling weak and/or strong resource monotonicity – Situations 13 and 14. Table S4.f. Pairs of allocations fulfilling weak and/or strong resource monotonicity – Situations 15 and 16. Table S5.a. Pairs of allocations fulfilling weak and/or strong severity monotonicity – Situations 1 and 5. Table S5.b. Pairs of allocations fulfilling weak and/or strong severity monotonicity – Situations 1 and 6. Table S5.c. Pairs of allocations fulfilling weak and/or strong severity monotonicity – Situations 1 and 7. Table S5.d. Pairs of allocations fulfilling weak and/or strong severity monotonicity – Situations 5 and 7. Table S6.a. Pairs of allocations fulfilling effectiveness monotonicity – Situations 1 and 3. Table S6.b. Pairs of allocations fulfilling effectiveness monotonicity – Situations 10 and 12. (PDF 446 kb) [file 12939_2017_611_MOESM3_ESM.pdf]

Allocating Health Care Resources:
A Questionnaire Experiment on the Predictive Success of Rules

Additional file 3: Supplements to Tables S4 to S6 in Additional file 2

This file contains further details on the calculation of hit rates and areas of prediction for each pair of situations in Tables S4 to S6.

Tables S4.a to S4.f:	Supplements to Table S4: Weak and strong resource monotonicity, in Additional file 2
Tables S5.a to S5.d:	Supplements to Table S5: Weak and strong severity monotonicity, in Additional file 2
Tables S6.a and S6.b:	Supplements to Table S6: Effectiveness monotonicity, in Additional file 2

Table S4.a: Pairs of allocations fulfilling weak and/or strong resource monotonicity – Situations 1 and 2

		Situation 2 (S_1 = 40, S_2 = 10, e_1:e_2 = 2:1, q = 60)							
	Allocations offered	(30,30)	(25,35)	(20,40)	(15,45)	(10,50)	(5,55)	(0,60)	Sum
Situation 1 (S_1 = 40, S_2 = 10, e_1:e_2 = 2:1, q = 30)	(30,0)	3	1	3	1	1	0	0	9
	(25,5)	1	0	1	0	0	0	0	2
	(20,10)	3	4	8	2	1	0	0	18
	(15,15)	8	6	13	6	0	0	0	33
	(10,20)	1	2	35	21	12	1	0	72
	(5,25)	1	1	4	8	11	1	0	26
	(0,30)	0	0	0	0	2	0	0	2
	Sum	17	14	64	38	27	2	0	N = 162

Note: S_1, S_2, status quo health levels of patients 1 and 2; e_1, e_2, effectiveness factors of patients 1 and 2; q, available units of treatment time.

Situations 1 and 2: Calculation of hit rates and areas of prediction

	# hits	Hit rate ($r = \frac{\#hits}{N}$)	Area of prediction (a)	Measure of predictive success ($m = r - a$)
Weak resource monotonicity	151	0.9321	28/49 = 0.5714	0.3607
Strong resource monotonicity	121	0.7469	20/49 = 0.4082	0.3388

Table S4.b: Pairs of allocations fulfilling weak and/or strong resource monotonicity – Situations 3 and 4

		Situation 4 (S_1 = 10, S_2 = 40, e_1:e_2 = 2:1, q = 60)						
	Allocations offered	(45,15)	(40,20)	(30,30)	(20,40)	(10,50)	(0,60)	Sum
Situation 3 (S_1 = 10, S_2 = 40, e_1 : e_2 = 2:1, q = 30)	(30,0)	1	2	1	0	0	0	4
	(25,5)	2	3	6	1	0	0	12
	(20,10)	1	12	49	5	0	0	67
	(15,15)	0	1	28	6	0	0	35
	(10,20)	1	2	9	27	1	0	40
	(5,25)	0	0	0	1	0	0	1
	(0,30)	0	0	1	0	0	0	1
	Sum	5	20	94	40	1	0	N = 160

Note: S_1, S_2, status quo health levels of patients 1 and 2; e_1, e_2, effectiveness factors of patients 1 and 2; q, available units of treatment time.

Situations 3 and 4: Calculation of hit rates and areas of prediction

	# hits	Hit rate ($r = \frac{\#hits}{N}$)	Area of prediction (a)	Measure of predictive success ($m = r - a$)
Weak resource monotonicity	158	0.9875	25/42 = 0.5952	0.3923
Strong resource monotonicity	148	0.9250	18/42 = 0.4286	0.4964

Table S4.c: Pairs of allocations fulfilling weak and/or strong resource monotonicity – Situations 8 and 9

		Situation 9 (S_1 = 30, S_2 = 15, e_1:e_2 = 2:1, q = 60)							
	Allocations offered	(35,25)	(30,30)	(25,35)	(20,40)	(15,45)	(10,50)	(0,60)	Sum
Situation 8 (S_1 = 30, S_2 = 15, e_1:e_2 = 2:1, q = 30)	(30,0)	1	0	1	0	0	0	0	2
	(25,5)	0	0	4	0	0	0	0	4
	(20,10)	3	4	5	1	0	0	0	13
	(15,15)	5	12	22	18	3	0	0	60
	(10,20)	1	2	6	39	15	1	0	64
	(5,25)	0	0	1	4	12	1	0	18
	(0,30)	0	0	0	0	0	0	0	0
	Sum	10	18	39	62	30	2	0	N = 161

Note: S_1, S_2, status quo health levels of patients 1 and 2; e_1, e_2, effectiveness factors of patients 1 and 2; q, available units of treatment time.

Situations 8 and 9: Calculation of hit rates and areas of prediction

	# hits	Hit rate ($r = \frac{\#hits}{N}$)	Area of prediction (a)	Measure of predictive success ($m = r - a$)
Weak resource monotonicity	160	0.9938	32/49 = 0.6531	0.3407
Strong resource monotonicity	151	0.9379	24/49 = 0.4898	0.4481

Table S4.d: Pairs of allocations fulfilling weak and/or strong resource monotonicity – Situations 10 and 11

		Situation 11 (S_1 = 40, S_2 = 20, e_1:e_2 = 3:1, q = 40)					
	Allocations offered	(20,20)	(15,25)	(10,30)	(5,35)	(0,40)	Sum
Situation 10 (S_1 = 40, S_2 = 20, e_1:e_2 = 3:1, q = 20)	(20,0)	3	4	1	0	0	8
	(15,5)	10	9	2	0	0	21
	(10,10)	9	21	26	1	0	57
	(5,15)	3	4	45	18	0	70
	(0,20)	0	0	0	3	0	3
	Sum	25	38	74	22	0	N = 159

Note: S_1, S_2, status quo health levels of patients 1 and 2; e_1, e_2, effectiveness factors of patients 1 and 2; q, available units of treatment time.

Situations 10 and 11: Calculation of hit rates and areas of prediction

	# hits	Hit rate ($r = \frac{\#hits}{N}$)	Area of prediction (a)	Measure of predictive success ($m = r - a$)
Weak resource monotonicity	151	0.9497	15/25 = 0.6000	0.3497
Strong resource monotonicity	95	0.5975	9/25 = 0.3600	0.2375

Table S4.e: Pairs of allocations fulfilling weak and/or strong resource monotonicity – Situations 13 and 14

		Situation 14 (S_1 = 25, S_2 = 5, e_1:e_2 = 3:1, q = 40)						
	Allocations offered	(25,15)	(20,20)	(15,25)	(10,30)	(5,35)	(0.40)	Sum
Situation 13 (S_1 = 25, S_2 = 5, e_1:e_2 = 3:1, q = 20)	(20,0)	7	4	5	4	1	0	21
	(15,5)	3	11	6	2	2	0	24
	(10,10)	4	17	5	5	2	0	33
	(5,15)	3	3	8	52	12	0	78
	(0,20)	0	0	0	2	3	0	5
	Sum	17	35	24	65	20	0	N = 161

Note: S_1, S_2, status quo health levels of patients 1 and 2; e_1, e_2, effectiveness factors of patients 1 and 2; q, available units of treatment time.

Situations 13 and 14: Calculation of hit rates and areas of prediction

	# hits	Hit rate ($r = \frac{\#hits}{N}$)	Area of prediction (a)	Measure of predictive success ($m = r - a$)
Weak resource monotonicity	145	0.9006	19/30 = 0.6333	0.2673
Strong resource monotonicity	115	0.7143	12/30 = 0.4000	0.3143

Table S4.f: Pairs of allocations fulfilling weak and/or strong resource monotonicity – Situations 15 and 16

		Situation 16 (S_1 = 55, S_2 = 15, e_1:e_2 = 3:1, q = 40)				
	Allocations offered	(15,25)	(10,30)	(5,35)	(0,40)	Sum
Situation 15 (S_1 = 55, S_2 = 15, e_1:e_2 = 3:1, q = 20)	(15,5)	8	1	0	0	9
	(10,10)	15	14	7	1	37
	(5,15)	8	38	54	1	101
	(0,20)	0	4	8	2	14
	Sum	31	57	69	4	N = 161

Note: S_1, S_2, status quo health levels of patients 1 and 2; e_1, e_2, effectiveness factors of patients 1 and 2; q, available units of treatment time.

Situations 15 and 16: Calculation of hit rates and areas of prediction

	# hits	Hit rate ($r = \frac{\#hits}{N}$)	Area of prediction (a)	Measure of predictive success ($m = r - a$)
Weak resource monotonicity	151	0.9379	10/16 = 0.6250	0.3129
Strong resource monotonicity	73	0.4534	6/16 = 0.3750	0.0784

Table S5.a: Pairs of allocations fulfilling weak and/or strong severity monotonicity – Situations 1 and 5

		Situation 5 (S_1 = 25, S_2 = 10, e_1:e_2 = 2:1, q = 30)							
	Allocations offered	(30,0)	(25,5)	(20,10)	(15,15)	(10,20)	(5,25)	(0,30)	Sum
Situation 1 (S_1 = 40, S_2 = 10, e_1:e_2 = 2:1, q = 30)	(30,0)	3	0	0	3	3	0	0	9
	(25,5)	0	0	1	0	0	1	0	2
	(20,10)	1	3	5	3	6	0	0	18
	(15,15)	1	3	6	13	9	1	0	33
	(10,20)	0	2	4	12	48	5	0	71
	(5,25)	0	0	0	3	17	6	0	26
	(0,30)	0	0	0	0	1	1	0	2
	Sum	5	8	16	34	84	14	14	N = 161

Note: S_1, S_2, status quo health levels of patients 1 and 2; e_1, e_2, effectiveness factors of patients 1 and 2; q, available units of treatment time.

Situations 1 and 5: Calculation of hit rates and areas of prediction

	# hits	Hit rate ($r = \frac{\#hits}{N}$)	Area of prediction (a)	Measure of predictive success ($m = r - a$)
Weak severity monotonicity	129	0.8012	28/49 = 0.5714	0.2298
Strong severity monotonicity	54	0.3354	21/49 = 0.4286	-0.0932
Contextual irrelevance of severity	75	0.4658	7/49 = 0.1429	0.3230

Table S5.b: Pairs of allocations fulfilling weak and/or strong severity monotonicity – Situations 1 and 6

		Situation 6 (S_1 = 40, S_2 = 25, e_1:e_2 = 2:1, q = 30)							
	Allocations offered	(30,0)	(25,5)	(20,10)	(15,15)	(10,20)	(5,25)	(0,30)	Sum
Situation 1 (S_1 = 40, S_2 = 10, e_1:e_2 = 2:1, q = 30)	(30,0)	2	0	1	3	2	1	0	9
	(25,5)	0	0	1	0	0	1	0	2
	(20,10)	0	0	4	9	4	1	0	18
	(15,15)	0	1	3	21	7	1	0	33
	(10,20)	0	2	2	20	40	8	0	72
	(5,25)	0	0	0	5	13	8	0	26
	(0,30)	0	0	0	0	1	1	0	2
	Sum	2	3	11	58	67	21	0	N = 162

Note: S_1, S_2, status quo health levels of patients 1 and 2; e_1, e_2, effectiveness factors of patients 1 and 2; q, available units of treatment time.

Situations 1 and 6: Calculation of hit rates and areas of prediction

	# hits	Hit rate ($r = \frac{\#hits}{N}$)	Area of prediction (a)	Measure of predictive success ($m = r - a$)
Weak severity monotonicity	123	0.7593	28/49 = 0.5714	0.1878
Strong severity monotonicity	48	0.2963	21/49 = 0.4286	-0.1323
Contextual irrelevance of severity	75	0.4630	7/49 = 0.1429	0.3201

Table S5.c: Pairs of allocations fulfilling weak and/or strong severity monotonicity – Situations 1 and 7

		Situation 7 (S_1 = 70, S_2 = 10, e_1:e_2 = 2:1, q = 30)				
	Allocations offered	(15,15)	(10,20)	(5,25)	(0,30)	Sum
Situation 1 (S_1 = 40, S_2 = 10, e_1:e_2 = 2:1, q = 30)	(30,0)	3	1	3	2	9
	(25,5)	0	1	1	0	2
	(20,10)	0	8	5	5	18
	(15,15)	7	12	12	2	33
	(10,20)	3	19	33	17	72
	(5,25)	0	3	15	8	26
	(0,30)	0	0	1	1	2
	Sum	13	44	70	35	N = 162

Note: S_1, S_2, status quo health levels of patients 1 and 2; e_1, e_2, effectiveness factors of patients 1 and 2; q, available units of treatment time.

Situations 1 and 7: Calculation of hit rates and areas of prediction

	# hits	Hit rate ($r = \frac{\#hits}{N}$)	Area of prediction (a)	Measure of predictive success ($m = r - a$)
Weak severity monotonicity	155	0.9568	22/28 = 0.7857	0.1711
Strong severity monotonicity	113	0.6975	18/28 = 0.6429	0.0547
Contextual irrelevance of severity	42	0.2593	4/28 = 0.1429	0.1164

Table S5.d: Pairs of allocations fulfilling weak and/or strong severity monotonicity – Situations 5 and 7

		Situation 7 (S_1 = 70, S_2 = 10, e_1:e_2 = 2:1, q = 30)				
	Allocations offered	(15,15)	(10,20)	(5,25)	(0,30)	Sum
Situation 5 (S_1 = 25, S_2 = 10, e_1:e_2 = 2:1, q = 30)	(30,0)	1	2	1	1	5
	(25,5)	0	3	3	2	8
	(20,10)	4	5	3	4	16
	(15,15)	3	10	17	4	34
	(10,20)	4	23	40	17	84
	(5,25)	1	1	6	6	14
	(0,30)	0	0	0	0	0
	Sum	13	44	70	34	161

Note: S_1, S_2, status quo health levels of patients 1 and 2; e_1, e_2, effectiveness factors of patients 1 and 2; q, available units of treatment time.

Situations 5 and 7: Calculation of hit rates and areas of prediction

	# hits	Hit rate ($r = \frac{\#hits}{N}$)	Area of prediction (a)	Measure of predictive success ($m = r - a$)
Weak severity monotonicity	155	0.9627	22/28 = 0.7857	0.1770
Strong severity monotonicity	123	0.7640	18/28 = 0.6429	0.1211
Contextual irrelevance of severity	32	0.1988	4/28 = 0.1429	0.0559

Table S6.a: Pairs of allocations fulfilling effectiveness monotonicity – Situations 1 and 3

		Situation 3 (S_1 = 10, S_2 = 40, e_1:e_2 = 2:1, q = 30) ***(relevant is patient 2: S_2 = 40, e_2 = 1)***							
	Allocations offered	(30,0)	(25,5)	(20,10)	(15,15)	(10,20)	(5,25)	(0,30)	Sum
Situation 1 (S_1 = 40, S_2 = 10, e_1:e_2 = 2:1, q = 30)	(30,0)	3	0	4	2	0	0	0	9
	(25,5)	0	0	2	0	0	0	0	2
	(20,10)	0	2	9	3	4	0	0	18
	(15,15)	0	4	7	9	11	1	1	33
(relevant is patient 1: S_1 = 40, e_1 = 2)	(10,20)	1	4	28	17	22	0	0	72
	(5,25)	0	2	16	5	3	0	0	26
	(0,30)	0	0	2	0	0	0	0	2
	Sum	4	12	68	36	40	1	1	N = 162

Note: S_1, S_2, status quo health levels of patients 1 and 2; e_1, e_2, effectiveness factors of patients 1 and 2; q, available units of treatment time.

Situations 1 and 3: Calculation of hit rates and areas of prediction

	# hits	Hit rate ($r = \frac{\#hits}{N}$)	Area of prediction (a)	Measure of predictive success ($m = r - a$)
Focus on lower effectiveness	78	0.4815	21/49 = 0.4286	0.0529
Focus on stronger effectiveness	41	0.2531	21/49 = 0.4286	-0.1755
Contextual irrelevance of effectiveness	43	0.2654	7/49 = 0.1429	0.1226

Table S6.b: Pairs of allocations fulfilling effectiveness monotonicity – Situations 10 and 12

	Allocations offered	Situation 12 (S_1 = 20, S_2 = 40, e_1:e_2 = 3:1, q = 20) ***(relevant is patient 2: S_2 = 40, e_2 = 1)*** (35,25)	(30,30)	(25,35)	(20,40)	(15,45)	Sum
Situation 10 (S_1 = 40, S_2 = 20, e_1:e_2 = 3:1, q = 20) ***(relevant is patient 1: S_1 = 40, e_1 = 3)***	(20,10)	3	3	2	0	0	8
	(15,15)	3	9	7	1	1	21
	(10,20)	1	12	36	9	0	58
	(5,25)	1	7	41	21	0	70
	(0,30)	0	0	3	0	0	3
	Sum	8	31	89	31	1	N = 160

Note: S_1, S_2, status quo health levels of patients 1 and 2; e_1, e_2, effectiveness factors of patients 1 and 2; q, available units of treatment time.

Situations 10 and 12: Calculation of hit rates and areas of prediction

	# hits	Hit rate ($r = \frac{\#hits}{N}$)	Area of prediction (a)	Measure of predictive success ($m = r - a$)
Focus on lower effectiveness	75	0.4688	21/49 = 0.4000	0.0688
Focus on stronger effectiveness	41	0.2563	21/49 = 0.4000	-0.1438
Contextual irrelevance of effectiveness	44	0.2750	7/49 = 0.2000	0.0750
